# The Impact of General Strike on Government Healthcare Delivery in Kerala State in India

**DOI:** 10.1155/2016/8096082

**Published:** 2016-05-03

**Authors:** Aasems Jacob, Heidi Weiss, Aju Mathew

**Affiliations:** ^1^Department of Internal Medicine, Monmouth Medical Center, 300 2nd Avenue, Long Branch, NJ 07740, USA; ^2^University of Kentucky, 800 Rose Street, CC448, Lexington, KY 40536, USA; ^3^University of Kentucky, 800 Rose Street, CC447, Lexington, KY 40536, USA

## Abstract

General strike (also known as hartal) is used as a mode of protest by organizations and political parties in India. It is generally thought that hartals negatively impact the healthcare delivery in a society. We used the Right to Information Act to obtain data from government health centers in Kerala state in India for four hartal days (H-day) and two control days (A-day and B-day) for each H-day, from sixteen health centers including 6 Community Health Center (CHC), 6 Secondary Health Center (SHC), and 4 Tertiary Health Center (THC). Data on emergency room visits was available for six HCs. 15 HCs had a statistically significant decrease in the number of outpatient visits on H-day. There was no difference in the number of outpatient visits between the two control days (A and B) in 15 HCs, suggesting the lack of a posthartal surge in visits. Median decrease in outpatient visits in CHCs, SHCs, and THCs was 50.4%, 59.5%, and 47.4%, respectively. Hartal did not impact the number of emergency room visits in 6 out of 7 health centers assessed. Our study identified a significant harmful impact on government healthcare delivery due to hartals in Kerala. These findings have major public health implications.

## 1. Introduction

Democracy permits freedom of expression, and it includes freedom to protest against grievances. Hartal is a form of general strike in democratic nations, where a political or social organization leads the people to protest against an unfavorable law or rule by shutting down activities in government offices, companies, schools, and colleges as well as disrupting public and private modes of transportation. The organization that calls for a hartal assures the general public and the government that the healthcare delivery through hospitals and clinics will not be impacted by the shutdown. However, hartal may negatively impact the ability of people to access healthcare by curtailing people's right to free movement and transportation. To the best of our knowledge, there are no studies that have evaluated the impact of hartal on healthcare delivery in India.

Kerala is a state in India that has attained high scores in human development indices, for instance, low infant mortality rate and high life expectancy rate that mirror a developed country [1]. It has a strong system of public healthcare delivery. However, due to the significantly strong presence of trade unions and a vibrant political system, Kerala has been affected by several hartals. Therefore, we aimed to investigate the impact of hartal on government healthcare delivery in Kerala.

## 2. Methods

In the state of Kerala, the government health system is designed in a referral pattern of community level, district level, and tertiary referral institutions [2]. The Community Health Centers (CHCs) provide basic healthcare facilities at the community level. The Secondary Health Centers (SHCs) comprise General or District Hospitals and Taluk Hospitals. The Tertiary Health Centers (THCs) comprise Medical College Hospitals. For the purpose of our study, we identified 25 government health centers including seven THCs, 10 SHCs, and 8 CHCs across Kerala [3]. The health centers were randomly selected from 14 districts of the state to avoid a regional bias.

We then identified seven statewide hartals between May 2014 and May 2015. We chose four hartal days for our study, after omitting three hartals that fall on Fridays, Saturdays, or Sundays (Table 1). We chose to obtain data on the number of outpatient visits for a total of twelve days, including four hartal days and eight nonhartal days. We chose to obtain data on the number of emergency room visits for a total of nine days, including three hartal days and six nonhartal days (excluded one hartal day due to incomplete data provided by several health centers).

In order to understand the variations in healthcare delivery, we chose two nonhartal days for each hartal day as a control. “A-day” was chosen as the day seven days prior to hartal (“H-day”). We aim to control for the variations in seeking healthcare that can be attributed to the day of the week by using data for A-day. We chose the day immediately after the H-day as “B-day” and will use it to evaluate a surge in outpatient visits following the hartal.

**Figure 1:**
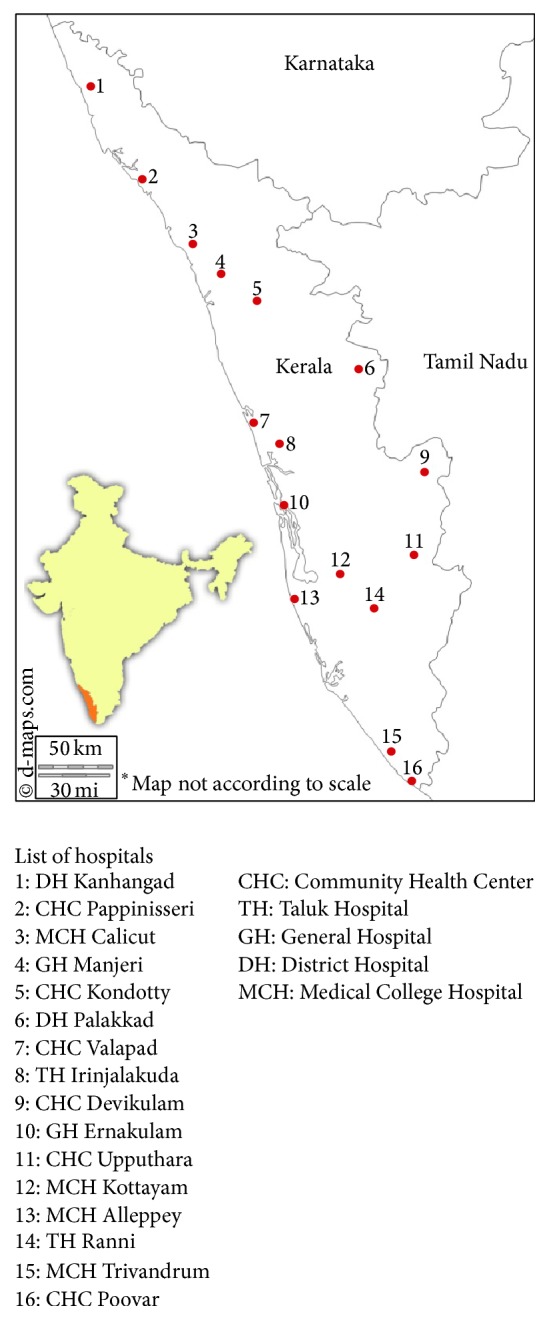
Locations of the health centers that contributed to the study.

Information on healthcare visits for these days was gathered through Right to Information (RTI) Act 2005 [4]. The Government of India passed the Right to Information Act in 2005 through which citizens can access information in government institutions following payment of a nominal fee. Nine health centers did not contribute to the study by either not responding to the request for information, rejecting the request, or providing incomplete information (Table 2). Information from sixteen health centers was used for the purpose of this study (Figure 1). Multiple inquiries and requests were necessary to obtain the data.

### 2.1. Statistical Analyses

Descriptive statistics including mean and standard deviation were calculated to summarize the number of outpatient visits for each health center on each of the A-day, H-day, and B-day along with bar graphs to depict % changes in number of outpatient visits compared to A-day. For each health center, a one-way analysis of variance (ANOVA) was employed to compare the number of outpatient visits across the 3 days of measurement along with pairwise comparisons between days. Test for homogeneity of variance across groups as well as normality assumptions for the ANOVA model was evaluated. Statistical analyses were performed using SAS 9.4.

## 3. Results

Outpatient visit data was available for 16 health centers, including 6 CHCs, 6 SHCs, and 4 THCs (Table 3). Emergency room visit data was available for 7 health centers including 5 SHCs and 2 THCs (Table 4). Our study identified a significant impact on healthcare delivery due to hartals in the state of Kerala.

**Figure 2:**
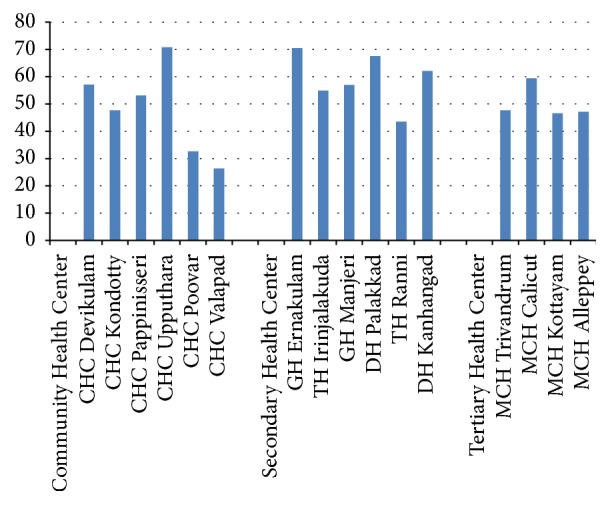
Decrease in outpatient visits on hartal days compared with A-day (%).

All CHCs except CHC Valapad had a statistically significant impact on the number of outpatient visits on H-day. There was no difference in the number of outpatient visits between the two control days (A and B), except for CHC Upputhara. Median decrease in outpatient visits in CHCs was 50.4% (range: 26.3–70.8) (Figure 2). All SHCs had a statistically significant impact on the number of outpatient visits on H-day. There was no difference in the number of outpatient visits between the two control days (A and B). Median decrease in outpatient visits in SHCs was 59.5% (range: 43.5–70.5). All THCs had a statistically significant impact on the number of outpatient visits on H-day. There was no difference in the number of outpatient visits between the two control days (A and B). Median decrease in outpatient visits in THCs was 47.4% (range: 47.1–59.4). Hartal did not impact the number of emergency room visits in 4 SHCs and 2 THCs except for TH Ranni.

## 4. Discussion

Our study found a significant impact of hartal on healthcare delivery in the state of Kerala. Based on information obtained from the health centers, the number of patients accessing healthcare in these institutions is significantly decreased on hartal days (H-day). The drop in outpatient visits was similar irrespective of the type of health center. Additionally, we found that the decrease in outpatient visits on hartal days is not correlated with an increase in outpatient visits on the subsequent day (B-day). These findings are further validated by the lack of a significant difference in outpatient visits between the two control days (A-day and B-day). We did not find a statistically significant impact on emergency room visits in six out of seven health centers that provided data. TH Ranni was an exception to this finding. Numerically, there was a decrease in number of emergency visits in all seven health centers.

Our study has few limitations. First, the study design is retrospective in nature. We used the Right to Information Act to obtain information on healthcare visits. There may be inherent errors in the reporting from these institutions. Since the data was collected without disclosure of the study aims, we believe that it represents the actual numbers seen in the health centers. Therefore, the use of RTI data may not have influenced the validity of our findings. Second, a major part of healthcare delivery in the state of Kerala is through the private sector [5]. Unfortunately, these hospitals do not fall under the purview of the RTI Act and therefore we were unable to include data from the private sector hospitals. However, based on our experience, the impact of healthcare delivery is expected to be more severe for the private institutions than the government hospitals which are always provided with extra security during these days. Third, we are unable to do further subgroup analysis due to lack of specific data, for instance, regarding the number of surgical cases in emergency room visits and the elective outpatient visits. These health centers rarely have previously scheduled outpatient appointments and therefore we do not expect that the drop in outpatient visits will be compensated by a surge in seeking healthcare over the subsequent weeks. Further prospective studies should evaluate the possibility of posthartal healthcare delivery in these health centers.

Nevertheless, we highlight a public health problem with a considerable impact on the health of Indian society. Prior studies have noted the economic burden of general strikes [6, 7]. To our knowledge, our study is the first to show the impact of hartal on public healthcare delivery. The key question is whether such an impact on accessing healthcare affects morbidity and mortality in societies. Several studies have shown that access to healthcare correlates with disease-specific outcomes in stroke, cardiovascular diseases, acute abdominal conditions, and road traffic accidents [8–13]. Based on these evidences and other anecdotal reports of death and injury due to hartals, we believe that hartals have a significant impact on the health of Indian society.

In conclusion, we believe that our study provides significant evidence for the public health burden of hartals in India. These findings may have considerable importance in policy-making. We hope that our study would raise a healthy public debate on the issue of healthcare impact of hartals and result in change in policies so that unnecessary mortality and morbidity can be avoided.

## Figures and Tables

**Table 1:** Selected hartal days.

Number	Date	Reason for hartal	Organization which called for the hartal
1	May 8, 2014 Thursday	Against Supreme Court decision on Mullaperiyar dam	Joint Action Council
2	September 2, 2014 Tuesday	Against alleged murder of RSS leader	RSS
3	January 27, 2015 Tuesday	Demanding resignation of finance minister on alleged charges of corruption	BJP
4	April 8, 2015 Wednesday	Various demands against laws and regulations of government	Motor Transport unions, Fisheries Coordination Committee, LDF Farmers Forum

**Table 2:** Health centers that did not contribute to the study.

Health center	Reason for noncontribution to the study
MCH Thrissur	Incomplete information (only provided total number of patients over the whole time period)
TH Punalur	Incomplete information (only provided total number of patients over the whole time period)
GH Sultanbatheri	Incomplete information (only provided total number of patients over the whole time period)
GH Thalasseri	Application rejected (citing the application fee was not enclosed)
CHC Nemmara	Incomplete information (visit data on some dates missing)
SAT Thiruvananthapuram	Application rejected twice (citing application fees need to be given in the form of a court fee stamp)
GH Kanjirappally	Discarded to avoid regional bias
GH Kollam	Application rejected (citing application fee was not enclosed)
CHC Sooranad	Application returned (address nonretrievable)

**Table 3:** Outpatient visits in health centers.

Health center	A-day (mean and standard deviation)	H-day (mean and standard deviation)	B-day (mean and standard deviation)	*p* value for the group	*p* value for A versus B	*p* value for A versus H	*p* value for B versus H
Community Health Center							
CHC Devikulam	55.5 (30.1)	23.8 (17.6)	71.0 (20.5)	0.049	0.37	0.09	0.02
CHC Kondotty	395.0 (134.0)	206.5 (40.4)	562.8 (138.2)	0.005	0.07	0.04	0.001
CHC Pappinisseri	446.3 (52.8)	198.0 (22.6)	467.5 (73.8)	<0.0001	0.59	<0.001	<0.001
CHC Upputhara	166.3 (51.6)	48.5 (12.4)	243.0 (46.5)	0.0003	0.03	0.002	<0.001
CHC Poovar	345.8 (48.2)	232.8 (13.6)	344.5 (49.9)	0.005	0.97	0.004	0.004
CHC Valapad	329.8 (59.8)	242.8 (48.3)	380.0 (102.5)	0.074	0.36	0.13	0.03

Secondary Health Center							
GH Ernakulam	1989.3 (451.6)	586.8 (210.1)	1897.0 (302.2)	0.0004	0.71	<0.001	<0.001
TH Irinjalakuda	532.3 (103.1)	239.8 (91.7)	642.8 (59.5)	0.0003	0.11	0.001	<0.001
GH Manjeri	2055.3 (470.5)	883.5 (437.4)	2246.8 (402.5)	0.0034	0.55	0.004	0.002
DH Palakkad	988.8 (154.0)	321.3 (136.3)	921.3 (102.1)	<0.0001	0.49	<0.001	<0.001
TH Ranni	408.0 (107.4)	230.3 (122.5)	515.0 (88.0)	0.0134	0.19	0.04	0.004
DH Kanhangad	767.3 (139.7)	290.8 (89.4)	896.0 (177.0)	0.0004	0.23	0.001	<0.001

Tertiary Health Center							
MCH Trivandrum	3490.8 (316.6)	1825.0 (776.5)	2990.5 (911.7)	0.025	0.35	0.01	0.046
MCH Calicut	3869.3 (916.6)	1572.5 (601.2)	2975.0 (922.6)	0.01	0.16	0.003	0.04
MCH Kottayam	1429.8 (104.6)	763.3 (209.5)	1419.5 (217.3)	0.0009	0.94	<0.001	<0.001
MCH Alleppey	2099.0 (605.6)	1108.3 (499.7)	1757.8 (211.9)	0.042	0.33	0.015	0.08

**Table 4:** Emergency room visits in health centers.

Health center	A-day (mean and standard deviation)	H-day (mean and standard deviation)	B-day (mean and standard deviation)	*p* value for the group	*p* value for A versus B	*p* value for A versus H	*p* value for B versus H
Emergency room visits							
GH Ernakulam	179.3 (19.1)	167.0 (11.5)	171.7 (19.9)	0.69	0.61	0.41	0.75
TH Irinjalakuda	189.0 (24.8)	162.0 (38.3)	162.7 (45.0)	0.62	0.42	0.40	0.98
GH Manjeri	298.0 (41.3)	253.3 (57.5)	300.3 (90.9)	0.64	0.97	0.44	0.42
DH Palakkad	163.3 (33.1)	142.7 (33.5)	139.0 (52.1)	0.74	0.49	0.55	0.92
TH Ranni	153.7 (32.0)	93.0 (16.1)	160.7 (33.0)	0.048	0.78	0.04	0.03
MCH Alleppey	451.0 (158.5)	342.7 (16.0)	334.3 (104.6)	0.41	0.24	0.27	0.92
MCH Trivandrum	467.3 (14.0)	396.7 (33.1)	441.0 (70.9)	0.24	0.51	0.11	0.28
